# Genomic characterization of *Bordetella pertussis* in South Africa, 2015–2019

**DOI:** 10.1099/mgen.0.001162

**Published:** 2023-12-20

**Authors:** Fahima Moosa, Mignon du Plessis, Michael R. Weigand, Yanhui Peng, Dineo Mogale, Linda de Gouveia, Marta C. Nunes, Shabir A. Madhi, Heather J. Zar, Gary Reubenson, Arshad Ismail, M. Lucia Tondella, Cheryl Cohen, Sibongile Walaza, Anne von Gottberg, Nicole Wolter

**Affiliations:** ^1^​ Centre for Respiratory Diseases and Meningitis, National Institute for Communicable Diseases of the National Health Laboratory Service, Johannesburg, South Africa; ^2^​ School of Pathology, Faculty of Health Sciences, University of the Witwatersrand, Johannesburg, South Africa; ^3^​ Division of Bacterial Diseases, National Center for Immunization and Respiratory Diseases, Centers for Disease Control and Prevention, Atlanta, Georgia, USA; ^4^​ Department of Science and Technology/National Research Foundation, Vaccine Preventable Diseases, University of the Witwatersrand, Johannesburg, South Africa; ^5^​ South African Medical Research Council Vaccines and Infectious Diseases Analytics Research Unit, Faculty of Health Sciences, University of the Witwatersrand, Johannesburg, South Africa; ^6^​ Wits Infectious Diseases and Oncology Research Institute, Faculty of Health Sciences, University of the Witwatersrand, Johannesburg, South Africa; ^7^​ Department of Pediatrics and Child Health, Red Cross Children’s Hospital, Cape Town, South Africa; MRC Unit on Child and Adolescent Health, University of Cape Town, Cape Town, South Africa; ^8^​ Rahima Moosa Mother & Child Hospital, Department of Pediatrics & Child Health, Faculty of Health Sciences, University of the Witwatersrand, Johannesburg, South Africa; ^9^​ Sequencing Core Facility, National Institute for Communicable Diseases of the National Health Laboratory Service, Johannesburg, South Africa; ^10^​ Department of Biochemistry and Microbiology, Faculty of Science, Engineering and Agriculture, University of Venda, Thohoyandou 0950, South Africa; ^11^​ Institute for Water and Wastewater Technology, Durban University of Technology, Durban 4000, South Africa; ^12^​ School of Public Health, Faculty of Health Sciences, University of the Witwatersrand, Johannesburg, South Africa

**Keywords:** *B. pertussis*, whole genome sequencing, Illumina sequencing, PacBio sequencing, vaccine antigen genes, genome structural characterization

## Abstract

Pertussis remains a public health concern in South Africa, with an increase in reported cases and outbreaks in recent years. Whole genome sequencing was performed on 32 *Bordetella pertussis* isolates sourced from three different surveillance programmes in South Africa between 2015 and 2019. Genome sequences were characterized using multilocus sequence typing, vaccine antigen genes (*ptxP*, *ptxA*, *ptxB*, *prn* and *fimH*) and overall genome structure. All isolates were sequence type 2 and harboured the pertussis toxin promoter allele *ptxP3*. The dominant genotype was *ptxP*3*-ptxA*1*-ptxB2-prn*2*-fimH*2 (31/32, 96.9 %), with no pertactin-deficient or other mutations in vaccine antigen genes identified. Amongst 21 isolates yielding closed genome assemblies, eight distinct genome structures were detected, with 61.9 % (13/21) of the isolates exhibiting three predominant structures. Increases in case numbers are probably not due to evolutionary changes in the genome but possibly due to other factors such as the cyclical nature of *B. pertussis* disease, waning immunity due to the use of acellular vaccines and/or population immunity gaps.

## Full-Text

### Data availability

The genome sequences for 32 South Africa *B. pertussis* isolates are available on NCBI, organized under BioProject accession number PRJNA929342.

## Introduction

Pertussis disease caused by *Bordetella pertussis* is a public health concern in South Africa with peaks observed every 3–5 years [1–3]. Until the start of the coronavirus disease 2019 (COVID-19) pandemic, cases had continued to increase globally over the last 20 years, including in South Africa. The rise in cases in South Africa could be attributed to pathogen adaptation or waning immunity as a result of the switch to the acellular pertussis vaccine in 2009 [4, 5] and/or improved awareness and diagnosis by clinicians and laboratories within the country [6, 7].

Acellular pertussis vaccines may be composed of up to four *B. pertussis* purified protein antigens (fimbrial antigens, pertactin, filamentous haemagglutinin and pertussis toxin). One of the factors thought to be contributing to the global rise in *B. pertussis* infections is pathogen adaption, which has increased (predominantly within the genes encoding vaccine antigens) since the introduction of acellular vaccines [8, 9]. This has resulted in the circulation of strains that have diverged from strains used in the vaccine [10–13]. Changes in the pertussis toxin promoter (*ptxP*) region have been observed, with the majority of isolates now harbouring the novel *ptxP*3 allele which replaced the *ptxP*1 allele [9, 14]. Strains harbouring the *ptxP*3 allele first emerged in the 1980s and have been shown to result in increased production of pertussis toxin *in vitro* [10]. Mutations within pertactin, filamentous haemagglutinin and pertussis toxin genes have also been described [15–18].

In 2009, South Africa replaced the whole-cell pertussis vaccine with a hexavalent vaccine which protects against diphtheria, tetanus, pertussis, poliomyelitis and *Haemophilus influenzae* type b, and is given to infants as part of the routine immunization programme [Pentaxim (2009–2014) replaced by Hexaxim (2015 to present); Sanofi Pasteur] [19]. Both Pentaxim and Hexaxim contain pertussis toxin and filamentous haemagglutinin antigens and do not contain pertactin (https://www.sanofi.com). In 2019, according to WHO/UNICEF, the coverage for the first and third dose of the pentavalent vaccine in South Africa was 84 and 77 %, respectively [20]. Between 2013 and 2018, among systematically tested individuals hospitalized with pneumonia, annual *B. pertussis* incidence was 17 cases per 100 000 population [1]. In addition, in 2018 and 2019, clusters of pertussis disease were observed in several provinces in South Africa [21].

*B. pertussis* was previously regarded as monomorphic due to lack of observable diversity using available molecular methods. Traditional typing methods such as multilocus sequence typing (MLST) or typing of genes encoding vaccine antigens provide poor discriminatory power [10, 22]. Since the introduction of whole genome sequencing (WGS), typing methods that include parts of and/or the entire genome, such as analysis of the accumulation of SNPs within the genome, whole genome MLST and core genome MLST, have added to the currently available techniques that can be used to classify circulating strains with more resolution compared to the traditional molecular methods [10, 22, 23]. More recently, the combination of long- and short-read WGS technologies have enabled a better understanding of genome arrangement patterns and genomic structural diversity amongst circulating isolates [8, 13, 17]. Using this methodology, unique genomic structures have been described in the USA [13], New Zealand [17] and India [24].

*B. pertussis* can undergo structural changes caused by alterations in their genome either by insertions, deletions or gene rearrangements, resulting in the circulation of strains that have diverged from vaccine strains [10, 13, 25, 26]. Strains not expressing the vaccine antigens pertactin and filamentous haemagglutinin have been described in the USA following the use of acellular vaccines [16, 25, 27]. Following acellular vaccine introduction, pertactin-deficient strains identified in France were shown to be as virulent as pertactin-expressing strains [28]. These data highlight the potential for the emergence of other vaccine escape mutants following the use of acellular pertussis vaccines and emphasize the importance of genomic surveillance for monitoring *B. pertussis* strain evolution in response to immune pressure. There are currently no data describing *B. pertussis* lineages in South Africa. Using WGS, we aimed to describe circulating *B. pertussis* genotypes and identify new or emerging immune escape mutations in South Africa.

## Methods

### Study population

*B. pertussis* isolates (*n*=32) were sourced from sentinel syndromic surveillance for severe respiratory illness (*n*=14), paediatric pertussis surveillance (*n*=11) and diagnostic samples (*n*=7). Prospective, active, sentinel pneumonia surveillance with *B. pertussis* testing was implemented in 2012 at sentinel sites in five of the nine provinces and enrols patients of all ages hospitalized with lower respiratory tract illness as well as neonatal sepsis or suspected sepsis in infants ≤3 months of age, irrespective of symptom duration [29]. Respiratory specimens (nasopharyngeal specimens and/or sputum) collected from patients are routinely tested for influenza virus, respiratory syncytial virus and *B. pertussis* by real-time PCR. Isolates included in this study were collected between 2015 and 2019.

Paediatric pertussis surveillance was conducted at the Chris Hani Baragwanath Academic Hospital (CHBAH) in Gauteng Province from January to December 2015 [30]. All infants aged <12 months who were hospitalized at CHBAH with lower respiratory tract infection or sepsis were approached for enrolment. Nasopharyngeal swabs were collected and tested for *B. pertussis* by culture and real-time PCR.

In addition, public sector clinicians in South Africa occasionally send respiratory specimens (nasopharyngeal specimens, sputum, tracheal aspirates) from patients of all ages to the National Institute for Communicable Diseases for *B. pertussis* diagnosis.

### *B. pertussis* culture and phenotypic characterization

All respiratory specimens were tested by real-time PCR for the detection of *B. pertussis *, *Bordetella parapertussis* and *Bordetella holmesii* as previously described [2, 31]. Since *B. pertussis* culture is not performed routinely at primary diagnostic laboratories, *B. pertussis* culture was attempted for all respiratory specimens immediately following a positive PCR, where the PCR cycle threshold value was ≤25. Specimens were plated on specialized charcoal agar (Diagnostic Media Products) and plates were incubated under aerobic conditions at 37 °C. Suspected *B. pertussis* colonies were confirmed using MALDI-TOF MS (Bruker) and real-time PCR [2, 31].

### DNA extraction, sequencing and genome assembly

Genomic DNA was extracted from cultures using the Gentra Pure Gene Yeast/Bact. kit (Qiagen) with the Qiagen protocol [25]. DNA was quantified using the Qubit 2.0 fluorometer (Invitrogen) and Qubit dsDNA BR assay kit and sequenced using both the Illumina MiSeq (Illumina) and the PacBio RSII (Pacific Biosciences) sequencing platforms. PacBio sequence data were used to identify genome structural variation. A combination of sequence data from both platforms was used when sequence data were incomplete or when results were not conclusive.

Genomic libraries for Illumina sequencing were prepared (paired-end libraries, 2×300 bp) using the Nextera XT DNA v3 MiSeq sequencing kit. Reads were trimmed at both ends to remove nucleotides with a quality score of <45 bp using CLC Genomics Workbench (v21.0.3; CLC Bio). The passed reads were *de novo* assembled using CLC Genomics Workbench. Illumina genomes were assessed for quality using QUAST (http://bioinf.spbau.ru/quast) on genome fraction, duplication ratio, total length, N50 value and number of contigs [13]. Acceptable Illumina data included: genome fraction >90 %; total length >3.75 Mb; N50 >5 kb; and contigs between 300 and 350. Libraries for PacBio sequencing were prepared using the SMRTbell template prep kit (Pacific Biosciences) and polymerase binding kit. PacBio sequencing data were *de novo* assembled, polished and reoriented to the common start position (creating a closed genome) using a CDC in-house Perl script as previously described [13]. The closed, assembled genomes were assessed on genome fraction, total length and N50 value [13]. Acceptable PacBio data included: genome fraction >90 %; total length >3.75 Mb; and N50 >5 kb. The reference genome used was *B. pertussis* E476 (vaccine strain: Tohama I – accession number: CP010964).

### Multilocus sequence typing and vaccine antigen gene typing

Assembled Illumina sequences were used to determine the *B. pertussis* MLST profile. Sequence type (ST) was assigned using the BIGSdb platform curated by the Institute Pasteur (https://bigsdb.pasteur.fr/bordetella) according to seven housekeeping genes (*adk*, *fumC*, *glyA*, *tryB*, *icd*, *pepA* and *pgm*).

Detection of the *ptxP*, *ptxA, ptxB*, *prn* and *fimH* genes, and mutations within the *prn* gene was performed using a high stringency Basic Local Alignment Search (BLASTn) alignment (https://blast.ncbi.nlm.nih.gov/Blast) on assembled genomes as previously described [13, 25]. Assembled PacBio sequences were analyzed in a similar manner to supplement inconclusive results or incomplete Illumina sequence coverage.

### Phylogeny of *B. pertussis*

To determine the relationship between South African *B. pertussis* isolates and publicly available genomes of isolates from other countries, a tree was reconstructed by incorporating genomes from different countries (New Zealand, USA, Netherlands, Sweden, France, Norway, India, Japan and China) available on NCBI (accessed February 2023) that were representative of circulating strains (different ST’s) on each continent between 2012 and 2019, and also represented isolates with varying *ptxP* types (*ptxP1*, *ptxP2* and *ptxP3*). These included the reference genome *B. pertussis* E476 and the additional available vaccine strains from India [24] (Table S1, available in the online version of this article). A maximum-likelihood phylogenetic tree of 99 isolate genomes was reconstructed based on the concatenated alignment of the high-quality SNPs using CSI phylogeny (v1.4) [32] with additional tree annotation performed using iTOL (v6).

### Identification of genome structural variation

Genome structural variation was determined by aligning complete PacBio assemblies from 21 South African *B. pertussis* genomes to each other using progressiveMauve (v20150226) with default parameters [32]. The genomes were determined to be collinear if pairwise alignments included no observable inversions or gaps >1500 bp. Alignments were manually inspected and grouped into types based on each unique alignment. One representative sequence from each arrangement type (SA001, SA002 and SA003) as well as the singleton sequences (genome structures not shared with any other isolates) were further aligned, using progressiveMauve, to reported structures from the USA (CDC002, CDC010, CDC013, CDC046, CDC082 and CDC237) and New Zealand (NZ004, NZ005, NZ006, NZ007 and NZ008) [13, 17].

## Results

### *B. pertussis* isolates

A total of 32 *B. pertussis* isolates were available for sequencing. Isolates were obtained from children (*n*=25) and adults (*n*=7) from both surveillance as well as clinical diagnostics. All 32 isolates were cultured from sporadic *B. pertussis* cases. There were no known epidemiological links between the *B. pertussis* cases.

From 2015 to 2019, a total of 17 097 hospitalized individuals were enrolled into sentinel pneumonia surveillance of whom 228 (1.3 %) tested PCR positive for *B. pertussis *. We attempted culture on 108 of these PCR-positive samples (Ct≤25). A total of 14 samples (12.9 %, 14/108) yielded a *B. pertussis* culture. Positive cultures were isolated from individuals ranging from <3 months of age to 64 years of age, and 50.0 % (7/14) of cases were people positive for human immunodeficiency virus (HIV) (Table S2).

On the pertussis paediatric surveillance platform, from 1 January to 31 December 2015 *B. pertussis* PCR positivity was 2.3 % (42/1839) [30]. A *B. pertussis* culture was obtained from 26.2 % (11/42) of PCR-positive samples. Cases with positive culture were infants aged <3 months, and the majority were unvaccinated (72.7 %, 8/11) and HIV-unexposed (63.6 %, 7/11).

A total of 770 respiratory samples were received from 2015 to 2019 for *B. pertussis* diagnostic testing, of which 12.1 % (93/770) tested positive for *B. pertussis* by PCR. A total of 41 of these positive samples were further cultured and seven samples yielded a *B. pertussis* culture (7/41, 17.1%), all of which were from infants aged <3 months.

### Molecular characterization of *B. pertussis*

Using WGS data, all 32 *B. pertussis* isolates were characterized as ST2 (allelic profile: *adk*1, *fumC*1, *glyA*1, *tryB*3, *icd*1, *pepA*1, *pgm*1). Isolates belonged to the *ptxP*3 lineage and all genomes harboured an intact pertactin gene with no mutations. The vaccine antigen profile *ptxP*3-*ptxA*1-*ptxB*2-*prn*2-*fimH*2 was identified in 31/32 (96.9 %) isolates (Table S2). Overall, based on molecular characterization, all vaccine antigen genes were wild-type. One isolate (SA12) had an SNP within the *fimH*2 allele (A245G synonymous mutation) which was further classified as *fimH*2-1. This mutation was confirmed using both the Illumina and PacBio sequence data. The vaccine antigen profile for this isolate was *ptxP*3-*ptxA*1-*ptxB*2-*prn*2-*fimH*2-1.

### Phylogeny of *B. pertussis*

**Fig. 1.**
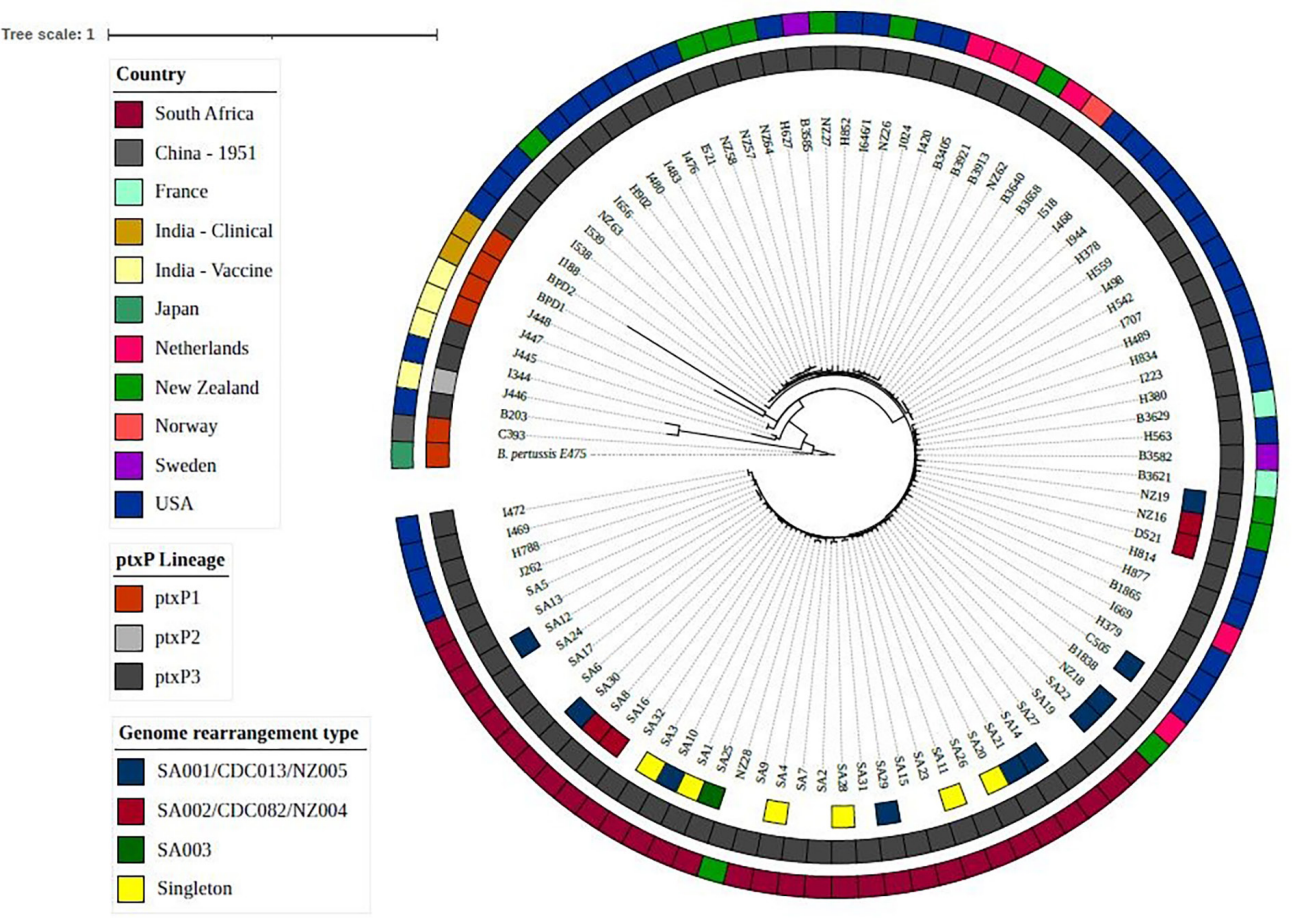
Maximum-likelihood phylogenetic tree for South African *B. pertussis* isolates and isolates from other geographical locations (2012–2019) based on the concatenated alignment of SNPs of the whole genome. The tree was rooted using the *B. pertussis* E476 reference (vaccine strain: Tohama I). The inner ring of the figure indicates the genome rearrangement type for South African isolates, the middle ring indicates the *ptxP* lineage and the outer ring indicates the country of isolation.

All South African isolates clustered within the *ptxP*3 clade and were phylogenetically distinct from *B. pertussis* E476 and other global strains (Fig. 1). There were two clusters (Cluster 1: seven South Africa+one New Zealand; and Cluster 2: six South Africa+four USA) where South African isolates clustered with isolates from the USA and New Zealand. Despite having identical ST and vaccine antigen profiles, based on SNPs, the South African isolates were genetically distinct from isolates from other countries (with the exception of the one New Zealand and four USA strains), clustering closely to each other within the tree. In addition, looking at the South African isolates only, there was no geographical clustering of strains and no temporal association of strains was noted.

### *B. pertussis* genome structural variation

**Fig. 2.**
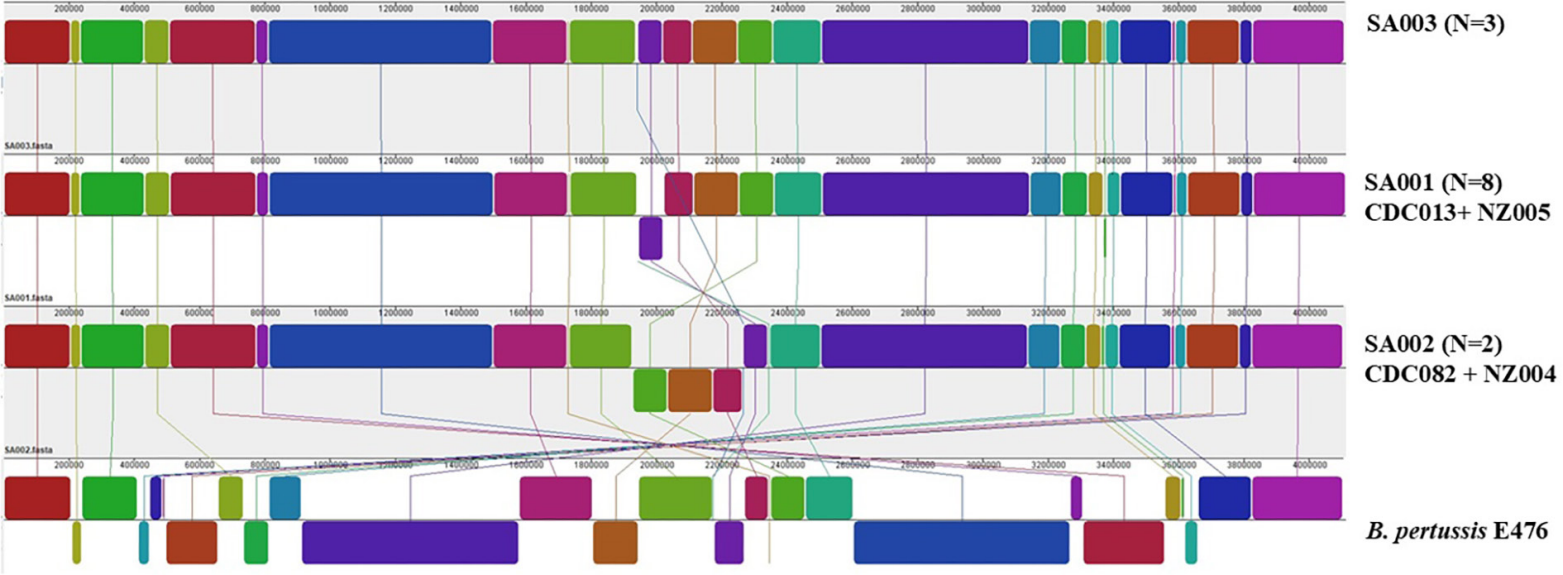
Genome organization and re-arrangement patterns amongst South African *B. pertussis* isolates collected in 2015–2019 in relation to reference E476 (*n*=13). Each coloured block represents a region of the genome called a localized co-linear block. A localized co-linear block below the central black line indicates an inversion event.

Of the 32 isolates sequenced using the PacBio, 21 (65.6 %) yielded closed genome sequences that could be analyzed for structural variation. The remaining 11 genomes yielded incomplete assemblies with more than one contig and thus could not be used to assess genome structure. Thirteen isolates (13/21, 62 %) could be grouped into three distinct arrangement types [SA001 (*n*=8), SA002 (*n*=2) and SA003 (*n*=3)] based on genome patterns, whilst eight isolates demonstrated unique (‘singleton’) structures distinct from the other arrangement types (Fig. 2 and Table S2). Genomic differences between the arrangement types were due to inversions centred around the replication terminus of the *B. pertussis* genome and differed from reference strain E476 (Tahoma I). The inversions were flanked by the *B. pertussis* multi-copy insertion sequence IS*481*. Comparing the genome structures to isolates from the USA and New Zealand, we found that SA001 was identical in structure to CDC013/NZ005 and SA002 matched structure CDC082/NZ004. In addition, one South African singleton structure (SA20) matched genome structure CDC046/NZ48. There was no concordance between SNP clusters and genome structural clusters amongst South African, USA and New Zealand isolates that shared the same genome structures (Fig. 1). Amongst the SA001/CDC013/NZ005 isolates, we found <18 SNP differences amongst the South African isolates within the structure and <25 SNP differences amongst South African isolates compared with the USA isolates and the New Zealand isolates. In addition, there were no SNP differences amongst the South African isolates within the SA002/CDC082/NZ004 cluster but when the South African isolates were compared to USA and New Zealand isolates we found <18 SNP differences amongst all the isolates.

## Discussion

In this *B. pertussis* genomic characterization study, isolates from South Africa, collected over a 5 year period, were genetically homogeneous using MLST. The dominant vaccine antigen genotype in all isolates except one was *ptxP3-ptxA1-ptxB2-prn2-fimH*2 and there was no evidence of mutations within the genes encoding vaccine antigens. WGS provided additional discriminatory power and identified three distinct genome structures.

Using MLST, South African *B. pertussis* isolates were the same sequence type (ST2) that has been dominant globally since the late 1990s [33]. In addition, all harboured the same pertussis toxin promoter allele, *ptxP*3. Globally, strains harbouring the *ptxP*3 allele first emerged in the 1980s [10]. Subsequently, over 90 % of globally circulating strains now harbour this allele [34, 35].

In other countries, mutations in vaccine antigen genes of *B. pertussis* appear to be more common since the introduction of the acellular vaccine, presumably due to vaccine pressure [8–10, 36]. In our setting, only one isolate contained a SNP within the vaccine antigen *fimH*2 gene. In addition, the South African isolates had an intact pertactin gene and no other mutations were identified within the other vaccine antigen genes. In the USA, pertactin-containing vaccines were introduced in the 1990s. From 2000 to 2013 in the USA, there was a rapid increase in pertactin-deficient *B. pertussis* isolates, resulting in non-expression of the gene [37]. In Australia the acellular pertactin-containing vaccine was introduced in the 1990s and strains lacking the pertactin gene were first detected between 2008 and 2012 [38]. Additionally, isolates collected between 2012 and 2017 showed spread of pertactin-deficient strains as well as an isolate that was negative for filamentous haemagglutinin. In Spain, between 1986 and 2018 a total of 342 *B. pertussis* isolates were collected for molecular characterization [39]. Of the 342 isolates, 27 % (93/342) were pertactin-deficient and all of these pertactin-deficient isolates were collected after the introduction of the acellular vaccine. Mutations within pertactin and other vaccine antigen genes were not detected among South African isolates, probably due to the formulation of the acellular pertussis vaccine (Hexaxim; Sanofi Pasteur), which only contains the pertussis toxin and filamentous haemagglutinin antigens, whilst many other countries are using vaccines containing the pertactin antigen as well as two or more other antigens. Maternal vaccination has been recommended for use in South Africa in 2024. The formulation of the vaccine to be used for maternal immunization includes pertactin and fimbriae, so ongoing genomic surveillance following the introduction of this vaccine will be important to monitor potential mutations within vaccine antigen genes amongst circulating *B. pertussis* strains in South Africa.

Using the traditional typing method of MLST and vaccine antigen profile data, our isolates represented a single clone. However, both methods rely on a very limited set of genes in the genome, limiting their discriminatory power. Determining the complete genomic structures of *B. pertussis* isolates using long read sequencing technology is relatively new and was first described in 2017 [13]. Using this method, WGS provided additional resolution and identified three distinct genome structures amongst 13 isolates. A combination of both MiSeq and PacBio sequence data proved to be advantageous as we used both data sets to create complete genomes from which we were able to define the genome structure. Studies have shown marked differences amongst *B. pertussis* genome structures despite having an otherwise monomorphic profile (based on other genome characterization methods). During two state-wide epidemics in the USA in 2010–2012, whole genome data were useful in identifying 16 distinct genome structures from 31 isolates that were otherwise monomorphic based on other typing techniques, and all differed from the vaccine strains [25]. In addition, Weigand *et al*. provided some evidence of phenotypic diversity amongst genome structures by showing the expansion of ‘cluster 01 (same genome structure)’ within the USA by analysing data from pre-2000 to 2016 [40]. Similarly, in New Zealand, WGS data showed 19 different genome rearrangements amongst 66 isolates [17]. An integrated comparative genomics analysis using isolates collected from the Czech Republic between 2008 and 2012 showed that circulating *B. pertussis* has substantial variation in genome organization and form separate phylogenetic clusters based on time of collection (historical isolates vs. currently circulating isolates) [41]. When using proteomics, they found that genome rearrangements resulted in changes in gene order, orientation or large deletions, which affected transcriptomic profiles in *B. pertussis *. Results support the authors’ assumption that genomic rearrangements might affect global expression profiles and phenotypic diversity in *B. pertussis *. Based on the above-mentioned data, genome structures/rearrangements could be useful signatures of genome evolution. Within our study, two of the South African genomic structures (SA001 and SA002) identified were shared with structures identified in isolates collected in the USA and New Zealand, showing that these structures are not unique to South Africa. In addition, comparing the SNP differences amongst the South African, USA and New Zealand isolates within shared structures, we see that isolates within the same structures are genetically similar (<25 SNP differences). However, based on this analysis we are unable to determine ancestral relationships amongst the South African isolates and isolates from the USA and New Zealand with shared genome structures. Previous studies have demonstrated the low SNP density amongst global *B. pertussis* isolates, making it difficult to deduce geographical clustering and/or source attribution [10, 42]. Similarly, Weigand *et al*. have also shown that isolates of *B. pertussis* with shared genome structures are not automatically similar based on SNPs [40]. There are few data available that describe the importance of structural variation and more studies are required to fully understand the prevalence of arrangement type, and associations with geographical location, phenotype or strain fitness.

This study has some limitations that need to be considered. To our knowledge, there are no earlier *B. pertussis* genomic data available from South Africa so we are unable to track changes in the genomes over a longer period of time. The absence of earlier data also limits conclusions pertaining to the origin of currently circulating strains. In addition, South Africa changed to the acellular vaccine in 2009, and isolates sequenced in this study were collected between 2015 and 2019. This time period may therefore be too short to observe the effects of the change in vaccine. We had a small sample size of 32 genomes, representing <5 % of *B. pertussis* PCR-positive samples. Therefore, these data are not representative of all *B. pertussis* causing disease in the country and we may have missed detecting novel or emerging lineages or vaccine antigen mutations. *B. pertussis* is a fastidious organism that is difficult to culture in the laboratory, requires specialized culture media that have a short shelf life, and has long incubation periods of 7–10 days, limiting its diagnostic utility. Diagnostic laboratories in South Africa do not routinely perform *B. pertussis* culture and rely predominantly on PCR testing for *B. pertussis* diagnosis. In addition, the low culture yield, incomplete geographical representation of sites and small sample numbers do not allow for confidence regarding potential temporal and geographical conclusions. The pneumonia surveillance programme focuses on respiratory viral detection and nasopharyngeal swabs are transported in viral transport medium which is not conducive to *B. pertussis* culture. Ideally, WGS of *B. pertussis* directly from clinical specimens needs to be optimized in our setting for future molecular characterization studies [9].

There are currently limited *B. pertussis* genomic data from Africa and no previously published data from South Africa. These findings now provide baseline genomic data for future studies within our country and in the global context. Although limited and spanning a short time period of 5 years, we show that circulating *B. pertussis* strains in South Africa do not yet harbour mutations in vaccine antigen genes, as has been observed in other countries. Increases in pertussis cases may be due to other factors such as the cyclical nature of *B. pertussis* disease, waning immunity due to the use of acellular vaccines that also do not protect against *B. pertussis* carriage and/or population immunity gaps, and these warrant closer examination within our region. Our findings have implications to help guide recommendations for improved vaccination strategies in low- and middle-income countries.

## Supplementary Data

Supplementary material 1
